# Remote follow-up using patient-reported outcome measures in patients with chronic kidney disease: the PROKID study – study protocol for a non-inferiority pragmatic randomised controlled trial

**DOI:** 10.1186/s12913-019-4461-y

**Published:** 2019-09-04

**Authors:** Birgith Engelst Grove, Per Ivarsen, Annette de Thurah, Liv Marit Schougaard, Derek Kyte, Niels Henrik Hjøllund

**Affiliations:** 1 AmbuFlex/WestChronic, Occupational Medicine, University Research Clinic, Aarhus University, Herning, Denmark; 2 Department of Nephrology, Aarhus University Hospital, Aarhus, Denmark; 3 Department of Rheumatology, Aarhus University Hospital, Aarhus, Denmark; 4 Department of Clinical Medicine, Aarhus University, Aarhus, Denmark; 5 Health Research Methods, Centre for Patient-Reported Outcomes Research (CPROR), Institute of Applied Health Research, University of Birmingham, Birmingham, UK; 6 Department of Clinical Epidemiology, Aarhus University Hospital, Aarhus, Denmark

**Keywords:** Chronic kidney disease, Outpatient clinic, Patient-reported outcome measures, Ambulatory care, Randomised controlled trial

## Abstract

**Background:**

Outpatient care is steadily changing from hospital consultations to other platforms, such as phone consultation and online virtual clinics. It is prudent to maintain quality of care with such initiatives. Currently, patients with chronic kidney disease (CKD) have frequent scheduled visits, but it may be possible to optimise the frequency of hospital consultations using information from patient-reported outcome (PRO) questionnaires filled in at home (PRO-based follow-up). This approach may provide a more individually tailored follow-up based on actual needs for clinical attention. We aimed to evaluate the effectiveness of the quality of care, use of resources and patient outcomes associated with PRO-based follow-up in patients with CKD.

**Methods:**

This study is a pragmatic, non-inferiority, randomised controlled trial in outpatients with CKD (Grove BE et al., Qual Life Res 27: S143, 2018). Newly referred patients with an estimated glomerular filtration rate (eGFR) of ≤40 ml/min 1.73m^2^ will be randomised to either:

1. PRO-based remote follow-up
2. PRO-based telephone consultation
3. Usual outpatient follow-up (control group)

In the two intervention groups, a diagnosis-specific PRO questionnaire completed by the patient at home will substitute for usual outpatient follow-up visits. The PRO questionnaire will in part be used as a screening tool to identify patients in need of outpatient contact and to identify focus areas. Responses from the questionnaire will be processed according to a disease-specific algorithm and assigned green, yellow or red status according to patients’ needs.

The primary outcome will be loss of renal function evaluated by eGFR. Secondary outcomes are 1. Clinical outcomes, including initiation of acute dialyses, hospitalisation and mortality, 2. Utilisation of healthcare resources and 3. PRO measures, primarily quality of life (Euroqol EQ-5D) and illness perception (Brief Illness Perception Questionnaire (BIPQ).

**Discussion:**

Benefits and possible drawbacks of the PRO-based follow-up will be evaluated. If PRO-based follow-up proves non-inferior to usual outpatient follow-up, a reorganisation of routine clinical practice in nephrology outpatient clinics may occur. Further, results may impact other patient groups with chronic conditions attending regular follow-up.

**Trial registration:**

ClinicalTrials.gov identifier NCT03847766 (Retrospectively registered on January 23, 2019).

## Background

### Chronic kidney disease

Lifestyle and a growing elderly population in Denmark have increased the number of patients with chronic diseases to approximately one million Danes, with growing healthcare expenditure as a consequence [1]. In order to meet this demand, the healthcare system is changing from inpatient activity towards a greater extent of outpatient activity [2].

Also, the number of patients with chronic kidney disease (CKD) is increasing, affecting approximately 10% of the adult population [3]. In daily clinical practice, patients with CKD are monitored with regular blood samples and hospital visits according to national recommendations [4]. Outpatient care aims to delay progression and complications of CKD, such as cardiovascular disease, bone and mineral disease and malnutrition [5]. Traditional follow-up is based on an assessment of renal function and a brief dialogue with the patient. The estimated Glomerular Filtration Rate (eGFR) is accepted as the best overall measure of kidney function [6]. This method does not provide clinicians with detailed information regarding the impact of the disease on patient’s health-related quality of life (HRQOL), symptom burden and psychological function. Fatigue, loss of appetite, low self-rated health, loss of muscle mass and cognitive dysfunction are common symptoms in patients with renal insufficiency [7, 8]. Evaluation of these symptoms in the clinic environment relies mostly on subjective information from the patient, which emphasises the importance of using the patient’s voice. Evidence suggests that immediate intervention in advanced CKD can prevent/delay the progression of the disease and the subsequent need for initiating dialysis, reduce the risk of associated cardiovascular disease, and prevent events including myocardial infarction and stroke [9]. Effective management of advanced CKD, however, relies on the timely detection of deterioration, which can be a major challenge between scheduled outpatient visits. Therefore, it is often difficult to identify clinical deterioration unless a patient self-reports. Information about health-related symptoms may be collected systematically using patient-reported outcome (PRO) measures.

### Patient reported outcome (PRO) measures

A PRO is defined as “A measurement based on a report that comes directly from the patient about the status of a patient’s health condition without interpretation of the patient’s response by a clinician or anyone else” [10]. A PRO focuses on the source of information and emphasises the patient perspective and is often collected through questionnaires. The use of PROs in clinical practice is becoming increasingly common, and can be used as a tool to support communication between the clinician and patient, and to inform clinical decision-making [11, 12]. Furthermore, use of PROs has been shown to improve patient management and help patients to feel more involved and empowered in decisions around their care [13, 14].

**Fig. 1:**
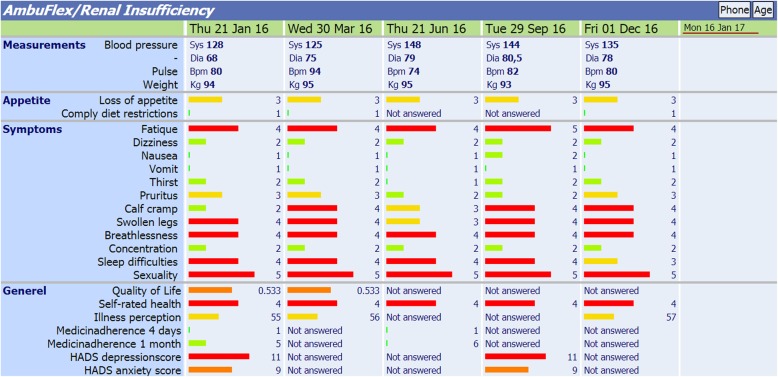
Screen capture of the clinicians’ overview in the nephrology clinics accessed from the electronic health record of Central Denmark Region. Colours of the bars indicate the severity of the symptoms reported by the patient. A red or orange bar indicates a self-reported problem, a yellow bar some problem, and a green bar indicates no problems. Copyright by AmbuFlex with permission to reuse. Note: Labels were translated from Danish

In Denmark, PRO systems are being implemented on a large scale based on national initiatives [15–17]. One initiative called AmbuFlex is a generic web-administered PRO system used in outpatient follow-up. As of January 2019, AmbuFlex has been implemented in 23 patient groups at Danish hospitals nationwide [18, 19]. The overall aims of AmbuFlex are to improve quality of care and reallocate healthcare resources by using PRO measures as the basis for follow-up. The method is termed PRO-based remote follow-up and represents a new model of service delivery where the patients’ PRO measures are used as the very basis for outpatient follow-up [18, 19]. Patients’ longitudinal PRO-data are presented graphically to the clinician within the electronic health record system for use prior or during consultations (Fig. 1). Since 2013, the AmbuFlex system has been used for the outpatients under the care of the nephrology outpatient clinics at Aarhus University Hospital and Regional Hospital Central Jutland, Viborg. The web-based PRO system has been used as a dialogue support in the face-to-face consultations with the physician. When a PRO is used in daily clinical practice, diagnosis-specific questionnaires (PROs) filled in by the patient at home are used as a substitute for usual outpatient follow-up. Hence, patients only visit the outpatient clinic if there is a clinical need or if it is the patient’s wish [19].

Participation in PRO-based follow-up is decided by the individual clinician and the patients’ own preferences. At present, there is no knowledge of who is suitable for participation in PRO-based follow-up, and this issue has not been documented in other studies; however, health literacy (HL) seems to be a barrier to responding to PROs [20].

Recently, a randomised controlled trial using AmbuFlex, the TeRA study [21], investigated follow-up in patients with rheumatoid arthritis based on PRO-data compared to usual outpatient follow-up, and found no difference in disease control. The primary outcome was a change in the Disease Activity Score in 28 joints (DAS28) [21]. An ongoing study using AmbuFlex is investigating the effect of patient-initiated PRO-based follow-up among patients with epilepsy [22]. However, neither of these studies is evaluating whether PRO use can replace visits to the outpatient clinic. A Dutch pragmatic randomised controlled study on patients with inflammatory bowel disease (IBD) has investigated a telemedicine system: “My IBDcoach” using an electronic PRO as a tool to self-monitor and to prepare for outpatient visits. The system also included an electronic learning module and open communication with the clinicians. They found that the telemedicine intervention was safe and reduced outpatient visits and hospital admissions significantly compared to usual outpatient follow-up [23]. However, IBD is characterised by specific alert symptoms related to disease activity in contrast to a disease like CKD. Hence, it may be more complex to use PROs in a similar way in a population with CKD.

Patients with CKD attend outpatient clinics for kidney disease regularly for years and spend a lot of time on transportation to and from the hospital for consultations, yet a number of visits could be cancelled or postponed if the patients were stable and some essential information was available. As the patient group is heterogeneous with respect to comorbidity and cognitive difficulties, it may present a suitable patient population for a study describing advantages and barriers for the use of PRO-based follow-up. For patients with CKD, PRO-data may allow clinicians to monitor for symptom deterioration, facilitating the early detection of problems requiring attention and promoting timely intervention by the clinicians (e.g. advice aimed at aiding patient self-management or escalation of care) [8]. Such intervention may delay or prevent disease progression and the need for costly and invasive renal replacement treatment (RRT), and reduce hospitalisation and other adverse outcomes. Basch et al. found an overall survival associated with electronic patient-reported symptom monitoring vs. usual care based on follow-up from a randomised clinical trial among cancer patients. A potential mechanism of action was early responsiveness to patient symptoms, preventing adverse consequences. HRQOL improved among more participants in the intervention group than among those receiving usual care [24]. However, the evidence for using PROs as a basis for remote follow-up is scarce and not investigated in a CKD population. A randomised controlled trial is needed to evaluate the efficacy of the use of PRO in patients with advanced CKD to determine whether PRO-based follow-up should be implemented as routine care in clinical practice. The focus of this trial will be to evaluate whether PRO-based remote follow-up is at least as effective as usual outpatient follow-up in managing decline in renal function and maintaining patients’ quality of life.

## Methods

### Aim

The aim of this study is to compare the effectiveness of using PRO measures as follow-up with regard to clinical outcomes, the utilisation of resources, and patient-reported outcomes in three types of follow-up in a non-inferiority pragmatic randomised controlled trial.

### Hypothesis

#### Three a priori hypotheses will be tested

- Non-inferiority will be reached for both patients in the PRO-based remote follow-up and the PRO-based telephone consultations for the primary endpoint, loss of renal function (eGFR).
- Patients in the PRO-based remote follow-up and the PRO-based telephone consultations will perceive less deterioration in quality of life, but increased satisfaction and a gain a deeper understanding of illness perception than patients in usual care.
- Use of healthcare resources is less in the PRO-based remote follow-up and the PRO-based telephone consultations compared to usual follow-up

### Design

The PROKID study is a non-inferiority pragmatic multi-centre three-arm randomised controlled trial of adults with advanced CKD [25]. Participants will be randomised in a ratio of 1:1:1 to receive one of the following: (a) PRO-based remote follow-up, (b) PRO-based telephone consultations or (c) Usual outpatient follow-up visits. The study follows the SPIRIT checklist: Standard protocol for clinical trials [26, 27].

### Study population

Currently, patients with CKD under the care of a nephrologist are seen regularly in a hospital out-patient clinic. The frequency of follow-up varies depending on the particular patient’s needs and patient/clinician preferences, but the average frequency is around every 3 months. Current Danish clinical guidelines recommend that patients are referred to a nephrology outpatient clinic when their kidney function, measured as estimated glomerular filtration rate (eGFR), is approximately 30–40 mL/min/1.73m^2^ [4]. Referral is continuous, and approximately 30 patients are referred to the nephrology outpatient clinics each month. Newly referred patients under the care of the nephrology outpatient clinics at Aarhus University Hospital and Regional Hospital Central Jutland, Viborg will be recruited for this study.

#### Inclusion criteria

- EGFR between 10 and 39 mL/min 1.73m^2^
- Aged ≥18 years old
- Ability to answer a questionnaire and participate in the study

#### Exclusion criteria

- Patients unwilling to participate in PRO-based follow-up
- Patients who, in the opinion of the consenting professional, cannot speak, read or write Danish sufficiently well to complete the PRO questionnaire unaided
- Patients with impaired hearing or visual disabilities
- A projected risk of progression to end-stage renal disease within 12 months, determined from albumin/creatinine ratio > 1
- Patients with an eGFR ≤10 mL/min 1.73 m^2^ due to a projected risk of progression to end-stage renal disease
- Patients who have received (or have a scheduled date to receive) a kidney transplant
- A terminal illness that, in the opinion of the consultant assessing eligibility, is likely to lead to the death of the patient within 6 months of starting participation in the study
- Patients receiving chemotherapy, with end stage chronic obstructive lung disease, or with heart failure with an ejection fraction (EF) < 15%

### Randomisation

Randomisation will be provided by a secure online randomisation system. Patients will be randomised by the WestChronic software [19] in a ratio of 1:1:1, during the patient’s second visit to the clinic. Due to the nature of the intervention it is not possible to blind patients or clinicians involved in the trial. The clinicians are only capable of seeing the patients’ responses in the clinical PRO questionnaire and will be blinded for the patients’ answers given in the research questionnaire.

### Study timeline

**Fig. 2:**
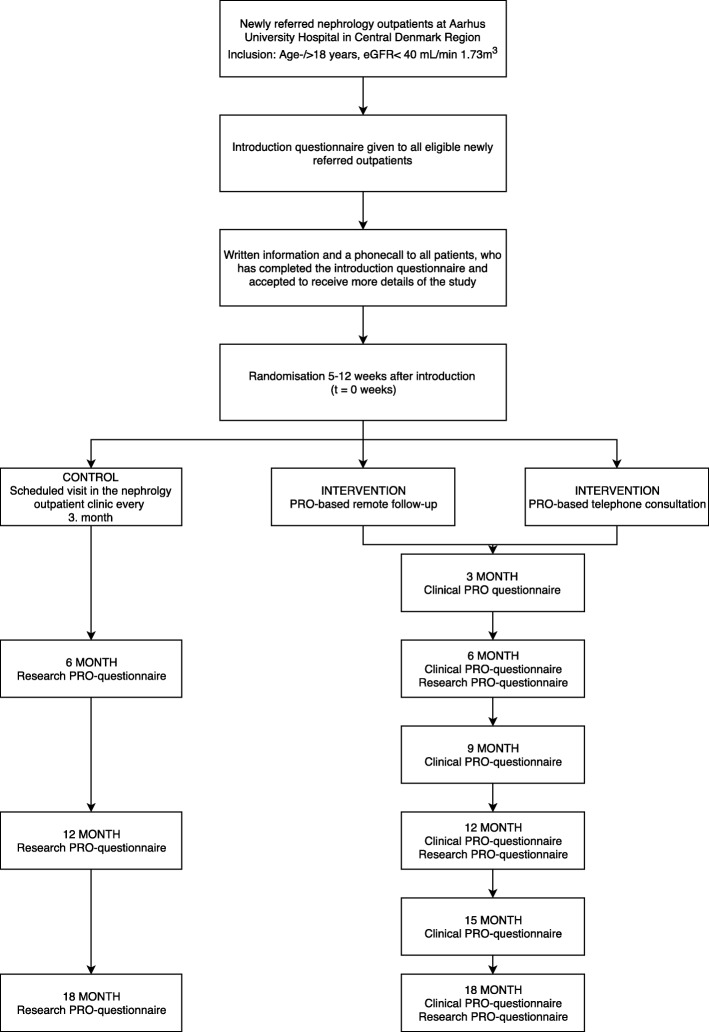
Flowchart following patients from introduction through inclusion and to final data collection

Inclusion and randomisation started in January 2019. Inclusion is expected to terminate in April 2020. Follow-up assessment will take place 6, 12 and 18 months after randomisation. Figure 2 presents the flowchart on inclusion of the participants. Baseline and follow-up PRO assessments are shown in Table 1.

**Table 1:** Complete overview of the questionnaires used in the PROKID project

Name	Introduction	Baseline	Clinical questionnaire	Research questionnaire
Purpose	Identify determinants for patients choosing PRO-based follow-up	Baseline characteristics	Using questionnaire as screenings tool and dialogue support	Repeated measures of characteristics
Respondents	ALL newly referred patients in renal outpatient clinic	ALL enrolled patients in PROKID study	Participants in PRO-based follow-up and in PRO-based telephone consultation arm	ALL patients in the three randomisation groups
Content	• Renal-specific items • SF-GH1 • HLQ^a^ (subscale 4,6,9) • Self-efficacy^b^ • PAM^c^(2 + 12) • Demographic • Consent (Yes/no)^h^	• Renal-specific domains • SF-GH1^d^ • EQ-5D^e^ • BIPQ^f^	Renal-specific domains • KDQOL-SF^g^ • EORTC • SF-GH1 • Need/wish for consultation • Free textbox	• Self-efficacy • EQ-5D • BIPQ • Patient involvement • Confidence • Satisfaction
No items	36	37	29	27

^a^ Health Literacy Questionnaire, ^b^ General Self-efficacy Scale (GSE), ^c^ Patient Activation Measure, ^d^ Short Form 36_GH1, ^e^ Euroqol Quality of Life, ^f^ Brief Illness Perception Questionnaire, ^g^ Kidney Disease Quality of Life Short Form,^h^ Consent to allow the researchers to obtain data on blood samples from the medical record

### PRO clinical questionnaire

The PRO clinical questionnaire will be used as a screening tool to identify patients in need of outpatient contact and to identify uraemia-related symptoms. It includes information on specific aspects of daily life with renal failure and comprises generic items from, e.g., SF-36 [28] and KDQOL-SF [29]. A recent systematic review favours this scale for use in a pre-dialysis population [30] as it has shown sound internal consistency and solid construct validity. In addition, clinically relevant kidney-specific items have been developed because of the lack of clinically relevant items. These items were identified by patients and clinicians as important factors in advanced CKD during interviews in the development phase prior to this study. The questionnaire is identically in the two intervention groups except for the question on “need of contact” in the PRO-based remote follow-up group. Modes of administration will be electronic and paper version questionnaires, which will be collected in the patients’ own homes. When a PRO instrument is used for decisions at the patient level in clinical practice, it is important that items are accepted as clinically relevant to both patients and clinicians [12]. The establishment of this type of validity, face validity, has been fundamental and has been ensured during the development process. The validation of the PRO questionnaire was based on the revised International Patient Decision Aid Standards (IPDAS) [31]. Content and face validity [19, 32] of the instrument have been assessed by a focus group interview comprising clinicians. In total, eight patients each participated in a one-to-one interview assessing comprehensibility and usability, relevance and deficits of the instrument. This resulted in a rephrasing of several items and new domains such as pain, gastrointestinal function and physical function were added to the questionnaire.

### PRO research questionnaire

The PRO research questionnaire covers a range of scales and items, which is shown in Table 1. Patients will answer this questionnaire at 0-, 6-, 12- and 18-month follow-up. Modes of administration will be electronic and paper version questionnaires collected in the patients’ own homes. The questionnaire will be obtainable in a Danish version and should always be completed by the patient, with possible assistance from a family member or friend. At baseline, the patient will answer the questionnaire on an iPad or on a paper version in the outpatient clinic. At the end of study or if a patient exits the study, he or she will be contacted and asked to answer the questionnaire. To minimise missing data, non-responders get two reminders after 4 and 7 days. The acceptable PRO assessment time window is 1 month. Patients’ responses to this questionnaire will not be available to the clinicians.

### Interventions

In the two intervention groups; PRO-based remote follow-up and PRO-based telephone consultations, usual outpatient follow-up visits will be replaced with diagnose-specific questionnaires (PROs) filled in by the patient at home. Patients in the two intervention groups will be contacted by e-boks, a secure electronic mailbox for all citizens in Denmark used to receive digital mail. Patients who do not have access to a computer will receive a paper version. Patients will be prompted to answer the PRO questionnaire either on-line or in a paper version at a scheduled time every 3 months. This interval forms the basis in both groups in order to ensure the same consultation interval as that of the usual follow-up (Fig. 2). A website was developed to collect PRO in the intervention groups [33]. This site allows patients to (a) answer the PRO questionnaire, (b) view their personal PRO data (previous PRO responses), (c) view information about the nephrology PRO questionnaire, specific questions and the study purpose. Patients who have access to the Internet are prompted to answer the questionnaire 1 week prior the appointed date for the PRO-based follow-up. Patients who prefer a non-electronic questionnaire will be prompted to answer the questionnaire 2 weeks prior to this date to secure enough time for completion and return of questionnaire. Physicians will review the patients’ responses to the PRO questionnaire via AmbuFlex, a generic web-administered PRO system integrated in the electronic health record system (Fig. 1). Responses are automatically scanned into the electronic health record, and the actual response for each item automatically results in a colour code (green, yellow or red) according to the given issue. The clinician at the nephrology clinic is responsible for handling the patients’ PRO responses and for giving feedback to the patients. To ensure that no patients are lost, non-responders will receive two reminders. Clinicians keep track of non-responders on a PRO alert list, and the non-responder will be contacted by a nurse.

Patients allocated to both intervention groups will have blood samples drawn at a local clinical practice or hospital. Patients will measure their own blood pressure and body weight at home, which they will be reminded of in the letter following the questionnaires. Patients will measure blood pressure on an identical instrument (Microlife BP A3L Comfort) and will follow a standardised guideline, meaning they will measure blood pressure three times at 2-min intervals after 5 min of rest before morning medication. In line with usual practice, all patients will be offered participation in kidney school, dietary guidance and a conversation with dialysis coordinators and transplantation nurses when needed, as local instruction prescribes.

#### Intervention group – PRO-based remote follow-up

Patients will receive a questionnaire at pre-specified intervals, every 3 months. The PRO questionnaire is used as a decision aid together with other available clinical data, such as biochemistry, blood pressure, body weight, to determine whether the patient needs a phone call or a face-to-face visit. Thus, patients only visit the outpatient clinic if there is a clinical need or they wish to [19]. The PRO questionnaire is also used to inform clinical decision making, i.e. dialogue support. All responses from the questionnaire are automatically processed according to an algorithm in green, yellow or red status. A red status indicates that the patient needs a contact with the clinic, and a physician will call the patient or schedule a visit in the outpatient clinic. Visits are scheduled immediately after the assessment of the questionnaire. A green status indicates that the patient has no current need of attention. The green status questionnaires are handled by a physician, who will send the patients feedback on the questionnaire and blood samples by secure electronic mail or a letter if the patient does not use e-boks. A date for the next PRO questionnaire is calculated by the system. A yellow status is given to the remaining patients. Based on an overview of the questionnaire and the patient’s blood samples, a physician decides whether this patient should have a telephone consultation or a face-to-face consultation. The patients can in all cases request a contact and thereby overrule any clinical decision of no visit is needed. Should need arise for acute consultations, the patients are asked to contact the clinic directly.

#### Intervention group – PRO-based telephone consultations

Patients receive a questionnaire every 3 months prior to a scheduled telephone consultation. The PRO questionnaire is used as dialogue support and problem-focusing aid during the telephone consultation (Fig. 1). The actual response for each item automatically results in a colour code (red, yellow or green). A red response indicates that the patient has a problem, a yellow colour indicates a potential problem, while a green colour indicates no problems. During the telephone consultation, the patient’s response to the questionnaire, results of blood tests as well as blood pressure and weight will be discussed.

#### Usual care (control group)

Patients in the control group will have usual scheduled outpatient follow-up visits at the hospital initiated by the physician every 3 months. Scheduled appointments will be pre-booked in the patients’ hospital charts at the initiation of study to ensure the pre-specified intervals. These patients do not use the clinical PRO questionnaire, but complete the research questionnaires. They have blood tests, blood pressure and weight measured in the outpatient clinic as usual practice.

### Outcomes

The effects of using PRO as the basis for follow-up will be evaluated with regard to three different aspects: clinical outcomes, health resource utilisation and patient-reported outcomes. Clinical outcomes include pivotal clinical quality measures (mortality, renal function, initiation of dialysis, hospitalisation). Health resource utilisation will be measured by number of contacts to the nephrology outpatient clinic (Table 2) and the patient-reported outcomes will be measured with regard to: quality of life, illness perception, patient activation, satisfaction and confidence towards the outpatient follow-up. An overview of primary and secondary outcomes and data sources is shown in Table 2.

**Table 2:** Primary and secondary outcomes, data sources and timeline for measurements

**Outcomes**	**Data source**	**Measurement/month**
**Clinical outcomes**		
Renal function (eGFR)	Electronic health record (EHR)	0,3,6,9,12,15,18
Mortality	Electronic health record (EHR)	6,12,18
Initiation of dialysis	Electronic health record (EHR)	6,12,18
Transplantation	Electronic health record (EHR)	6, 12,18
Hospitalisation	Electronic health record (EHR)	16,12,18
CKD biomarkers	Electronic health record (EHR)	0,3,6,9,12,15,18
**Resource utilisation**		
Number of face-to-face visits	Electronic health record (EHR)	6,12,18
Unexpected contacts	Electronic health record (EHR)	6,12,18
Phone contacts	Electronic health record (EHR)	6,12,18
**Patient-reported outcomes**		
Quality of life	Euroqol (EQ-5D 5 L)	0,6,12,18
General health	Short Form-36 (SF-36)	0,6,12,18
Illness perception	Brief Illness Perception (IPQ-B)	0,6,12,18
Self-efficacy	General Self-efficacy (GSE)	0,12,18
Patient involvement	AmbuFlex PRO questionnaire	6,12,18
Confidence, satisfaction	AmbuFlex PRO questionnaire	6,12,18

### Primary outcome

Loss of renal function measured as a change in eGFR is the primary outcome and is considered to be the most accurate single measurement for renal function assessment [34].

### The secondary outcomes

#### Clinical outcomes

Information on biochemical markers as eGFR, creatinine, albumin, haemoglobin and creatinine will be obtained from the electronic health record. Data on hospitalisation and admission days, transplantation and end-stage renal disease and mortality are obtained from The Hospital Business Intelligence Register in Central Denmark Region.

#### Resource Utilisation

Number of contacts includes all contacts to the outpatient clinic in the follow-up period, including face-to-face consultations, telephone consultations and additional contacts in the outpatient clinic. In addition, other hospital care contacts and hospitalisations will be obtained from the Hospital Business Intelligence Register in Central Denmark Region. Additional information on the nature of resource utilisation will be obtained and captured in REDCAP (Research Electronic Data Capture). REDCap is a secure, web-based application designed to support data capture for research studies [35].

#### Patient-reported outcomes

Quality of life (QOL) will be measured by EuroQol EQ-5D-5 L [36]. The EQ-5D-5 L questionnaire measures individual generic health status using five dimensions: mobility, self-care, usual activities, pain/discomfort, and anxiety/depression, and each dimension has five levels depending on severity of symptoms (1 no problems, 5 extreme problems) and a visual analogue scale. The scores can then be converted into a single index number. The index value will be used for calculation of quality-adjusted life years for a health economic analysis of the intervention, which will be conducted after completion of this study. The EQ-5D scale is favoured for use in patients with chronic kidney disease due to ease of use for patients [37]. Illness perception will be measured by the IPQ-B (Brief Illness Perception Questionnaire), which comprises five components. Each of these components holds a perception about one aspect of the illness, and together they provide an individual’s coherent view of an illness [38]. The IPQ-B is widely used and has sound psychometric properties [39].Self-efficacy will be measured by using the Danish version of General Self-Efficacy Scale (GSE) [40, 41]. GSE was designed to assess optimistic self-belief to cope with difficult demands in life [40, 41]. GSE includes 10 items with a response range from 1 “not at all true” to 4 “exactly true”. The GSE scale has been used in a range of research projects in different countries and populations, where it typically yielded sufficient psychometric properties [42]. Confidence, safety and satisfaction will be measured by using ad hoc items developed from the Danish PREM (Patient-Reported Experience Measure) questionnaire from the Danish Cancer Society.

All outcomes will be compared in the three groups and measured at baseline and after 6, 12 and 18 months of follow-up or at the time a patient leaves the study due to patient wish, death, transplantation or initiation of dialyses.

### Demographic, clinical and laboratory variables

Demographic factors such as gender, age, education and current employment status will be obtained from the introductory questionnaire. Clinical factors like comorbidity due to malignancy, diabetes mellitus, chronic obstructive airways disease, cerebrovascular disease, ischaemic heart disease and peripheral vascular disease are categorised according to the Charlson Comorbidity Index [43]. Biochemistry results from the clinical laboratory will be obtained from tests performed in accordance with the current standard of care.

### Other measurements

Prior to entering the study, an introduction PRO questionnaire (iPad or in paper form) will be handed out to all eligible patients to investigate whether patients participating in this study have higher health literacy skills and self-efficacy than those who decline to take part in the study or wish to drop out. Nutbeam defines HL as *“The personal, cognitive and social skills which determine the ability of individuals to gain access to, understand, and use information to promote and maintain good health*” [44]. Health literacy will be measured by using the Danish version of Health Literacy Questionnaire (HLQ) [45, 46]. The HLQ includes nine conceptually subscales with a total of 44 items. The HLQ has well-documented psychometric properties [46]. In this study, the HLQ subscales 4 (Social support for health), 6 (Ability to actively engage with healthcare providers) and 9 (Understand health information well enough to know what to do) will be used. Patient activation will be measured by two modified items from the Danish version of the Patient Activation Measures (PAM) [47]. During the analysis phase, we intend to use this data to evaluate our hypothesis that patients choosing to participate in this study may have higher health literacy skills and self-efficacy than those who decline to take part or who drop out.

### Statistical methods

The three randomisation groups will be compared in intention-to-treat analysis and per protocol as recommended in non-inferiority trials [48]. The primary comparison groups will be composed of those receiving usual care, versus each of the intervention groups separately. Analysis methods will be chosen according to the data type of the outcome under investigation.

- *Continuous endpoints (*e.g. *quality of life, estimated Glomerular Filtration Rate)*: These data will be summarised using means and standard deviations, with differences in means and 95% confidence intervals reported. Longitudinal plots of the data over time will also be constructed for visual presentation of the data. The primary analysis will be adjusted for covariates identified as potentially prognostic variables (e.g. sex and age) in a multiple linear regression model. A secondary unadjusted analysis will also be performed if covariate adjustment is not practical (e.g. due to low event rates).
- *Categorical (dichotomous) endpoints (*e.g. *PRO data, hospitalisation rates)*: For dichotomous secondary endpoints, the proportion of participants and percentages will be summarised between arms. Logistic regression will be used to produce estimates of relative risks and 95% confidence intervals.
- *Time-to-event endpoints (*e.g. *time to initiation of dialysis, mortality):* These endpoints will be modelled for each randomisation arm using survival analysis methods. Kaplan–Meier survival curves will be constructed for visual presentation of time-to-event comparisons. The primary analysis will be carried out using a Cox proportional hazards or an extended Cox model to include prognostic covariates. Hazard ratios will be produced with 95% confidence intervals.

Sensitivity analyses will be performed to test the robustness of the results in the presence of differences in the groups.

Sample size was estimated for the primary outcome loss of renal function (eGFR). From literature review, the non-inferiority limit for eGFR is determined to be 2.85 mL/min/1.73 m^2^/year [49]. Based on a literature review, the assumptions are an approximate loss in eGFR of 5 mL/min/1.73 m^2^/year, SD: 4 mL/min/1.73 [49]. Loss in eGFR is normally distributed. Given 80% statistical power and *p*-value of 0.05, we need 50 patients in each group, in total 150 patients. With an estimate of 250 incident patients seen yearly, 150 patients will be reached in an inclusion period of 1 year and 2 months due to the expected attrition [50].

### Ethics

The risks relating to participating are considered to be minimal as all clinical parameters are assessed by the physician. Adverse events reporting will therefore be limited to events which are required for outcome assessment. The reporting period will commence when the patient has been consented into the trial and ends at the end of follow-up. All patients approached will be given a short information sheet describing the study along with verbal information by a researcher during their first attendance at the clinic. This will allow time for potential participants to consider the information provided, discuss the trial with their family and friends, and decide whether to take part before consenting prior to the next visit. A detailed paper form information and consent form will be sent to the patients prior to their second visit at the outpatient clinic. Provided the patients feel they have had sufficient time to consider their potential involvement, consent may be sought at the second visit at the outpatient clinic. A project nurse will discuss the study with them in detail and give a comprehensive verbal explanation (explaining both the investigational and standard treatment options, and highlighting any possible benefits or risks relating to participation). Informed written consent will then be sought from the participants who agree to enter the study. All included patients will receive a card with contact information and are informed that study participating is entirely voluntary and they can withdraw from the study at any time without effecting future care. The Danish Data Protection Agency has accepted the study, no 1–16–02-873-17. In addition, the Danish Research Ethics Committee in Central Denmark Region was contacted and has stated that approval from the committee is not necessary for the present study. Written informed consent is obtained from all participants.

### Data security

All data activities in the study are documented and stored in the WestChronic web system [19]. This system is situated in a Server Park in Central Denmark Region using firewall and Threat Management Gateway. Backup is performed on weekly basis and all data transactions fulfil the requirements from the Danish Data Protection Agency.

## Discussion

The use of PROs in clinical practice has become increasingly common during recent years [51]. However, there is limited scientific evidence to guide clinical practice and only few international experiences to draw on. PROs often compose a supplement to the patient’s follow-up, but are infrequently used as the basis for outpatient follow-up. The focus of this trial will be to evaluate whether PRO-based follow-up is at least as effective as usual outpatient follow-up in managing decline in renal function and maintaining patients’ quality of life. Furthermore, we intend to identify the target patient group for using PRO-based follow-up, since we believe that not all patients will benefit from PRO-based follow-up. The results may help identifying patient groups that are suitable for PRO-based follow-up and thereby promote future PRO implementation.

The nephrology outpatient clinics at Aarhus University Hospital and Regional Hospital Central Jutland, Viborg have used PRO as a complimentary tool for consultations the last few years [52], and are therefore familiar with the clinical use of PRO. This could be an advantage regarding the understanding of the software and understanding the PRO overview in the electronic health record. On the other hand, it could disturb the intervention if the clinicians use PROs as a part of daily practice alongside this study. This has been taken into consideration during the initiation of this study. Ideally, all use of PROs in daily clinical practice should be limited during this study period; however, this would not be acceptable for the patients in the nephrology outpatient clinics since it is a part of their usual follow-up. Another concern is the willingness of the patients to participate in this study due to the 3-arm design. The study population consists mostly of fragile elderly patients whose preferences may be to attend regular follow-up at the hospital facing a physician. In the pilot phase, an assessment of the sample size will be done. Loss to follow-up is one of the main concerns in randomised controlled studies [53]. Loss of statistical efficiency can be overcome by increasing the number of participants in the study [54]. If a lower participation rate than expected occurs, we will consider: (i) opening up recruitment to patients already attending the clinic (i.e. those currently in follow-up); and (ii) inviting other nephrology centres to participate in the study.

Minimising workflow disruption is essential when implementing change in the organisation at a hospital [55, 56]. Prior to initiation of this study, workflow has been mapped to identify patient pathways and workflow in order to be able to describe the change in the organisation at the hospitals after implementing PRO in clinical practice. Implementation of PROs in patient care requires a change in the clinical practices of clinicians and health organisations. Dedicated communication, demonstration and training of the clinicians are important when implementing PRO in clinical practice [57, 58]. Education of staff personal has been prioritised during the preliminary phase, and a Standard Operating Procedure (SOP) form has been developed informing on how to handle the patients in the different randomisation arms and identify, e.g., when to call a patient in for a consultation with a dietician or dialysis coordinator. A review by Porter et al., presents a framework of the key requirements for the successful implementation of PROs in clinical practice, encompassing the instrument itself, purpose of the PRO, setting, feedback system, support to implementation (specific training for clinicians on the administration and interpretation of PROs) [51]. These issues and their recommendations have been a part of the design and arrangement of this study. Another potential challenge is clinician preferences. Each patient is assigned to a contact physician, which may contribute to a difference in how patients are handled. Several studies emphasise that the potential effect of the use of PROs is mediated by a modification of the behaviour of both patients and professionals [11, 56]. Information on who represents the responsible physician for the patient is recorded to ensure details on potential differences in treatment and decisions among physicians. Traditionally, outpatients are seen by a physician and a nurse. The nurse’s role is to obtain blood pressure and weight, but also to talk to the patient about his or her symptoms. A great concern from the nurse’ perspective is the lack of information patients in the intervention groups receive if they are only seen by a physician. To embrace this problem, a flowchart and a short-form manual showing the steps during the PRO-based consultation have been developed and handed out to the physicians. In the case record form (CRF) we will record the type of contact the patients have had with the clinic, describing nurse and physician roles and tasks. Two parallel qualitative studies will be scheduled by a qualitative researcher to focus on the perspectives of patients and clinicians. Qualitative interpretive descriptions of the patients’ perceptions of the influence the PRO-based follow-up had on their contact with the outpatient clinic and on their ability to handle the chronic disease will be examined. Likewise, a qualitative interpretive description of clinicians’ experiences with PRO-based follow-up will focus on their perceptions of this approach’s influence on their tasks, responsibilities and professional identity.

## Data Availability

Only the investigaters will have access to the data. A dissemination plan has been developed which is directed towards different stakeholders a) Patients with chronic diseases, b) Health professionals, c) Politicians, d) Patient organisations, e) The general public, f) Scientific circles.

## Abbreviations

CKD: Chronic kidney disease
CRF: Case record form
eGFR: Estimated glomerular filtration rate
EHR: Electronic health record
ePRO: Electronic patient-reported outcome
ESRD: End stage renal disease
GSE: General self-efficacy scale
HL: Health literacy
HLQ: Health literacy questionnaire
KDQOL-SF: Kidney Disease Quality of life Short Form
PREM: Patient-reported experience measure;
PRO: Patient-reported outcome
RCT: Randomised controlled trial
SD: Standard deviation
SF-36: Short Form Health Survey
SOP: Standard operation procedures
